# Immunological predictors of rhythmic transcranial magnetic stimulation (rTMS) efficiency in patients with treatment-resistant schizophrenia

**DOI:** 10.1192/j.eurpsy.2024.584

**Published:** 2024-08-27

**Authors:** S. A. Zozulya, A. N. Pomytkin, D. V. Tikhonov, V. G. Kaleda, T. P. Klyushnik

**Affiliations:** Mental Health Research Centre, Moscow, Russian Federation

## Abstract

**Introduction:**

Model and clinical studies demonstrate the efficiency of rhythmic transcranial magnetic stimulation (rTMS) in diseases associated with neuroinflammation. The therapeutic potential of rTMS is related to modulation of neuroplasticity in the CNS, activation of neurogenesis and reduction of neuroinflammatory processes. Presumably, one of the factors that determines the efficiency of rTMS can be the features of the immune status of patients.

**Objectives:**

To reveal the features of the spectrum of inflammatory markers in patients with treatment-resistant schizophrenia with different efficiency of rTMS.

**Methods:**

31 male patients aged 16 to 47 years (mean age 29.9 ± 8.4 years) with treatment-resistant schizophrenia who developed a first psychotic episode in adolescence (19-25 years) were examined. The course of rTMS was conducted for 3 weeks (15 sessions). Depending on the dynamics of clinical and psychometric parameters after the course of rTMS, the patients were divided into three groups: group 1 - with worsening of clinical condition (n=8); group 2 - without therapeutic effect (n=12); group 3 - with good therapeutic response (n=11). Before rTMS, leukocyte elastase (LE) and α1-proteinase inhibitor (α1-PI) activity, and the levels of autoantibodies to S-100B protein and myelin basic protein (MBP) in the plasma of patients were determined. The parameters of 18 healthy male donors without clinical signs of psychiatric and somatic pathology were used as controls.

**Results:**

All groups of patients were characterised by moderate and high levels of immune system activation, determined by a complex of inflammatory and autoimmune markers. At the same time, the high level of immune system activation in patients with low MTR efficiency was associated with low LE activity in plasma (within the reference range or below the lower limit - 200.6 (168.5- 220.3) nmol/min·mL), which was not consistent with the overall level of inflammation. This group of patients also showed high levels of antibodies to MBP compared to control values (p<0.05). The low LE activity can be explained by the transmigration of neutrophils from the blood to the brain due to a critical increase in the permeability of the blood-brain barrier, which is largely controlled by LE.

**Conclusions:**

The study confirmed the participation of immune mechanisms in the formation of therapeutic resistance in schizophrenia and revealed the characteristics of the spectrum of immune markers in patients with low efficiency of rTMS.

**Disclosure of Interest:**

None Declared

